# Expression of Four Methionine Sulfoxide Reductases in *Staphylococcus aureus*

**DOI:** 10.1155/2012/719594

**Published:** 2012-01-04

**Authors:** Kuldeep Singh, Vineet K. Singh

**Affiliations:** Microbiology and Immunology, Kirksville College of Osteopathic Medicine, A.T. Still University of Health Sciences, Kirksville, MO 63501, USA

## Abstract

*Staphylococcus aureus* possesses three MsrA enzymes (MsrA1, MsrA2, MsrA3) that reduce the S-epimer of methionine sulfoxide (MetO) and an MsrB enzyme that reduces R-MetO. The four *msr* genes are expressed from three different promoters. The *msrA1/msrB* genes are coexpressed. To determine the expression pattern of *msr* genes, three independent reporter strains were constructed where *msr* promoter was cloned in front of a promoterless *lacZ* and the resulting construct was integrated in the chromosome. Using these strains, it was determined that the *msrA1/B* expression is significantly higher in *S. aureus* compared to *msrA2* or *msrA3*. Expression of *msrA1/B* was highest during stationary phase growth, but the expression of *msrA2* and *msrA3* was highest during the early to midexponential growth phase. Expression of *msrA1/B* was induced by oxacillin and the expression of *msrA3* was upregulated by salt. Expression of *msrA2* remained unchanged under all tested conditions.

## 1. Introduction

*Staphylococcus aureus *is a versatile and aggressive pathogen responsible for causing a wide array of diseases ranging from mild skin infections such as folliculitis and carbuncles to life-threatening conditions such as bacteremia, pneumonia, and endocarditis [1–3]. In response to *S. aureus* invasion, the host immune system recruits neutrophils and macrophages that trigger the release of highly reactive oxygen species such as hydrogen peroxide, hydroxyl radical, singlet oxygen, and hypochlorous acid. These highly reactive species lead to the oxidation of DNA, lipids, and proteins [4].

In proteins, oxidative damage usually leads to a loss of protein function that disturbs cellular processes and metabolism [5, 6]. Such oxidative damage includes oxidation of the sulfur atom of methionine producing methionine sulfoxide. Oxidation of methionine results in two diastereomic forms of MetO (R-MetO and S-MetO). These two stereoisomeric MetO products are reduced by two different kinds of Msr enzymes—MsrA and MsrB. MsrA specifically reduces S-MetO, whereas MsrB specifically reduces R-MetO [7–9]. The MsrA and MsrB proteins share no homology at the primary sequence or structural levels. Orthologs of *msrA* and *msrB* are present in most organisms [10, 11]. In bacterial species, the genetic organization of *msrA* and *msrB* shows great variation. In numerous cases, *msrA* and *msrB* are transcribed as independent units and are located in different regions of the chromosome [12]. However, in many bacterial species, these two genes are located adjacent to each other and are cotranscribed [12–14]. In a few cases like *Neisseria*, *msrA* and *msrB* are transcriptionally fused [12, 15–17]. The copy number of *msrA* and *msrB *orthologs also varies widely in bacterial species. For example, *Escherichia coli* contains one copy each of *msrA *and *msrB*; *S. aureus,* 3 *msrA* and 1 *msrB*; *Vibrio cholerae,* 2 *msrA* and 3 *msrB*; all present in the chromosome [12, 15, 16]. *Rhizobium meliloti* possesses 3 *msrA* and 3 *msrB* genes and one of each is located on a plasmid [12]. Genetic redundancy is considered a strategy where organisms express specific genes under specific environments [12]. Additionally, MsrA and MsrB proteins are some of the most conserved proteins across prokaryotic and eukaryotic organisms suggesting important cellular functions [11, 12, 18]. In many studies, bacterial species deficient in Msr proteins have been shown to be sensitive to oxidative stress and in the cases of many pathogenic bacteria, their Msr knockout derivatives were shown to be attenuated in virulence [12, 15–17, 19–22].

Previously, in proteomic studies, upon exposure of actively growing *S. aureus *cells to oxacillin (a cell wall-active antibiotic), MsrA1 and MsrB proteins were observed to be produced in elevated amounts [23]. Subsequent gene fusion, Northern analysis, and transcriptional profiling experiments demonstrated an increased expression of *msrA1 *and *msrB *genes in the presence of oxacillin as well as several other cell wall-active antibiotics such as cephalothin, D-cycloserine, and bacitracin [24, 25].

It was speculated that the expression of different *msr *genes in *S. aureus *are regulated differently under different growth conditions as part of better survival strategy. Three independent reporter strains were constructed to test this assumption. In these reporter strains, the *msrA1/B*, *msrA2*, and *msrA3 *promoters were cloned in front of a promoterless *lacZ *gene and the resulting constructs were introduced into the chromosome of the *S. aureus *strain SH1000. Findings of this study suggest that the *msr* gene loci are differentially regulated in *S. aureus *that may have important physiological significances.

## 2. Materials and Methods

### 2.1. Bacterial Strains, Plasmids, Antibiotics, and Growth Conditions

The bacterial strains and plasmids used in this study are shown in Table 1. *S. aureus *cells were grown in tryptic soy broth (TSB) or tryptic soy agar (TSA), and *E. coli *cells were grown in Luria-Bertani broth (LB) or Luria-Bertani agar (LBA). Plasmids in *E. coli* cells were maintained by adding ampicillin at 100 *μ*g mL^−1^ and erythromycin at 15 *μ*g mL^−1^, when required. Overnight cultures of *S. aureus msr *reporter strains were prepared in the presence of erythromycin at 10 *μ*g mL^−1^.

### 2.2. DNA Manipulations

Plasmid DNA was isolated using the QIAprep Miniprep kit (Qiagen). All restriction and modification enzymes were purchased from Promega (Promega). PCR was performed using a Peltier Thermal Cycler-200 system (MJ research). DNA manipulations were carried out as described [26]. Oligonucleotide primers were obtained from Sigma Genosys.

### 2.3. Construction of an *msrA1/B* Promoter-*lacZ* Reporter Strain in *S. aureus* Strain SH1000

An *msrA1/B *promoter-*lacZ *reporter constructed previously in *S. aureus* strain RN450 [13, 23] was transferred to *S. aureus* strain SH1000 using a phage transduction procedure as described previously [13, 23] and the resulting construct was verified by PCR.

### 2.4. Construction of an *msrA2* Promoter-*lacZ* Reporter Strain in *S. aureus* Strain SH1000

Primers MsrA2P-1 (5′-TCTAGACAAGCAATTCACGTTG-3′) and MsrA2P-2 (5′-GAATTCCTTTCATTAGACCTTAG-3′) were used to amplify a 1281 bp DNA fragment using genomic DNA from *S. aureus* SH1000 as template. This amplicon represents the upstream and 8 nt of the 5′-end of the *msrA2 *gene. The amplicon was cloned in the correct orientation upstream of a promoterless *lacZ* gene of vector pAZ106 [27] and was introduced into the chromosome of *S. aureus* RN4220 by electroporation with selection on erythromycin. Phage 80*α* lysate of the resulting transformant was used to transduce the *msrA2 *promoter-*lacZ* fusion into strain *S. aureus* SH1000. A single copy integration of the *msrA2P*-*lacZ *in the chromosome was confirmed by Southern blot analysis.

### 2.5. Construction of an *msrA3* Promoter-*lacZ* Reporter Strain in *S. aureus* Strain SH1000

To construct the *msrA3 *reporter strain, two primers MsrA3P-1 (5′-GATCCAGCGACACCTCATCATTTGC-3′) and MsrA3P-2 (5′-GAATTCACCCTCCTGCTACATAAAC-3′) and genomic DNA from *S. aureus *strain SH1000 as template were used to PCR amplify a 1427 bp DNA fragment. The amplicon represents the upstream and 39 nt of the 5′-end of the *msrA3 *gene. The amplicon was cloned in the correct orientation upstream of the promoterless *lacZ* gene of vector pAZ106, introduced into the chromosome of *S. aureus* RN4220 by electroporation, and subsequently into strain *S. aureus* SH1000 using a phage transduction procedure. A single copy integration of the *msrA3P*-*lacZ *in the chromosome was confirmed by Southern blot analysis.

### 2.6. Growth Kinetics of *msr* Reporter Strains and Expression of *msr* Genes in *S. aureus*

Overnight cultures of *msr(A1/B)P-lacZ*, *msrA2P-lacZ, *and *msrA3P-lacZ* reporter constructs in *S. aureus *strain SH1000 were diluted 100-fold in fresh TSB with a flask-to-medium volume ratio of 6 : 1 and grown at 37°C with aeration at 220 rpm. Growth of these cultures was recorded by measuring OD_600_ every 30 min. The expression of individual *msr* gene locus was determined in these reporter constructs at different time points by assaying *β*-galactosidase using O-nitrophenyl-*β*-D-galactopyranoside (ONPG) as the substrate as described previously [13, 23].

### 2.7. Expression of *msr* Genes in *S. aureus* under Stress Conditions

Overnight cultures of *msr(A1/B)P-lacZ*, *msrA2P-lacZ, *and *msrA3P-lacZ* reporter constructs were diluted 100-fold in fresh TSB and allowed to grow at 37°C with aeration and shaking. At OD_600_ = 0.3, cells from 10.0 mL of culture were harvested and resuspended in 10.0 mL fresh TSB or TSB modified to impose a variety of different stress conditions. Antibiotic stress used oxacillin at 1.2 *μ*g mL^−1^; oxidative stress, H_2_O_2_ at 15 mM; alkaline stress, TSB at pH 9.0; acidic stress, TSB at pH 5.0; osmotic stress, TSB supplemented with 1.5 M NaCl. Cells were allowed to grow for 1 h. Subsequently, the bacterial cells were harvested and the *β*-galactosidase activity was measured. The *msr* reporter constructs pre-grown to OD_600_ = 0.3 were also exposed for 1 h to following chemical agents: diamide (5 mM), N-ethylmaleimide (0.05 mM), methyl viologen (paraquat, 20 mM), menadione (0.05 mM), cumene hydroperoxide (0.0125%), and sodium nitroprusside (5 mM). The bacterial cells were subsequently used to determine *β*-galactosidase activity.

### 2.8. Statistical Analysis

All results are reported as the mean ± SD of at least three trials. Data were analyzed with Dunnett's Method in one-way analysis of variance or with Student-Newman-Keuls Method in two-way analysis of variance using a statistical analysis computer programs (SigmaPlot for Windows, version 11.0, Systat Software, Inc.). Statistical significance was set at *P* < 0.05.

## 3. Results

### 3.1. Growth Kinetics of *msr(A1/B)P-lacZ*, *msrA2P-lacZ*, and *msrA3P-lacZ* Reporter Constructs in *S. aureus*

**Figure 1:**
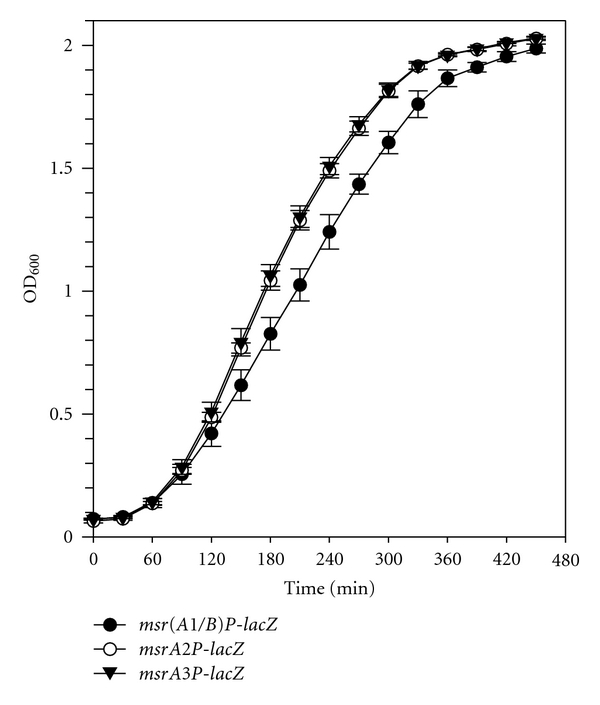
Growth comparison of the *msr(A1/B)P-lacZ*, *msrA2P-lacZ*, and *msrA3P-lacZ* reporter constructs in *S. aureus *strain SH1000. Growth was measured by recording OD_600_ periodically. Values indicate averages of data from three independent experiments ± standard deviation (SD). The *msr(A1/B)P-lacZ*, *msrA2P-lacZ*, and *msrA3P-lacZ* reporter strains are represented by closed circles, open circles, and closed triangles, respectively.

Fusion of individual *msrA1/B*, *msrA2*, and *msrA3 *promoters with the promoterless *lacZ* gene and their subsequent integration into *S. aureus* chromosome was verified by PCR and Southern blot analysis (data not shown). Subsequently, the growth rates of the above constructed reporter strains were analyzed to see if this promoter-*lacZ* integration in the staphylococcal chromosome caused any impact on growth. The results showed that the *S. aureus msrA2* and *msrA3* reporter strains grew almost at the same rate, whereas the *msrA1/B* reporter strain demonstrated a slightly slower growth rate (Figure 1).

### 3.2. Expression of *msr* Genes during Various Growth Stages in *S. aureus*

**Figure 2:**
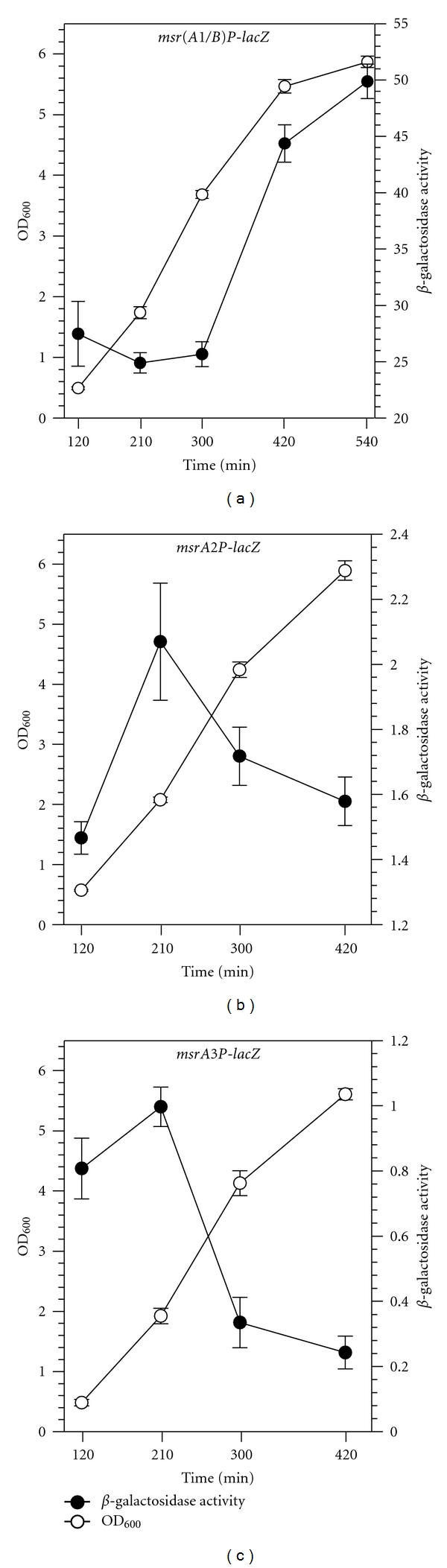
*β*-galactosidase activity levels in *msr(A1/B)P-lacZ *(a), *msrA2P-lacZ *(b), and *msrA3P-lacZ *(c) reporter strains during different stages of growth under standard growth conditions. Growth and *β*-galactosidase activity were measured at different time points spectrophotometrically. For precise OD_600_ determination, the late-stage cultures were diluted appropriately to bring cell density in measurable range of the spectrophotometer. OD_600_ is indicated by open circles, and *β*-galactosidase activity (OD_420_) is indicated by closed circles. Values indicate averages of data from three independent experiments ± standard deviation (SD).

Bacterial cells from the cultures of *msr(A1/B)P-lacZ*, *msrA2P-lacZ, *and *msrA3P-lacZ* reporter strains were collected during different stages of growth to investigate if the expression of these genes is growth phase dependent. In these studies, the expression of the *msrA1/B *gene locus was low during the early- and mid-exponential growth phases, but was significantly higher during the late exponential and stationary growth phases (Figure 2(a)). Expression of *msrA2 *and *msrA3* genes, on the other hand, was more pronounced during the early- and mid-exponential phases of growth and was much lower during the stationary growth phase (Figures 2(b) and 2(c)). These experiments also showed that the *msrA2* and *msrA3* genes are expressed at significantly lower levels compared to the expression of the *msrA1/B* locus at all stages of growth (Figures 2(a), 2(b), and 2(c)).

### 3.3. Expression of *S. aureus msr* Genes under Stress Conditions

**Figure 3:**
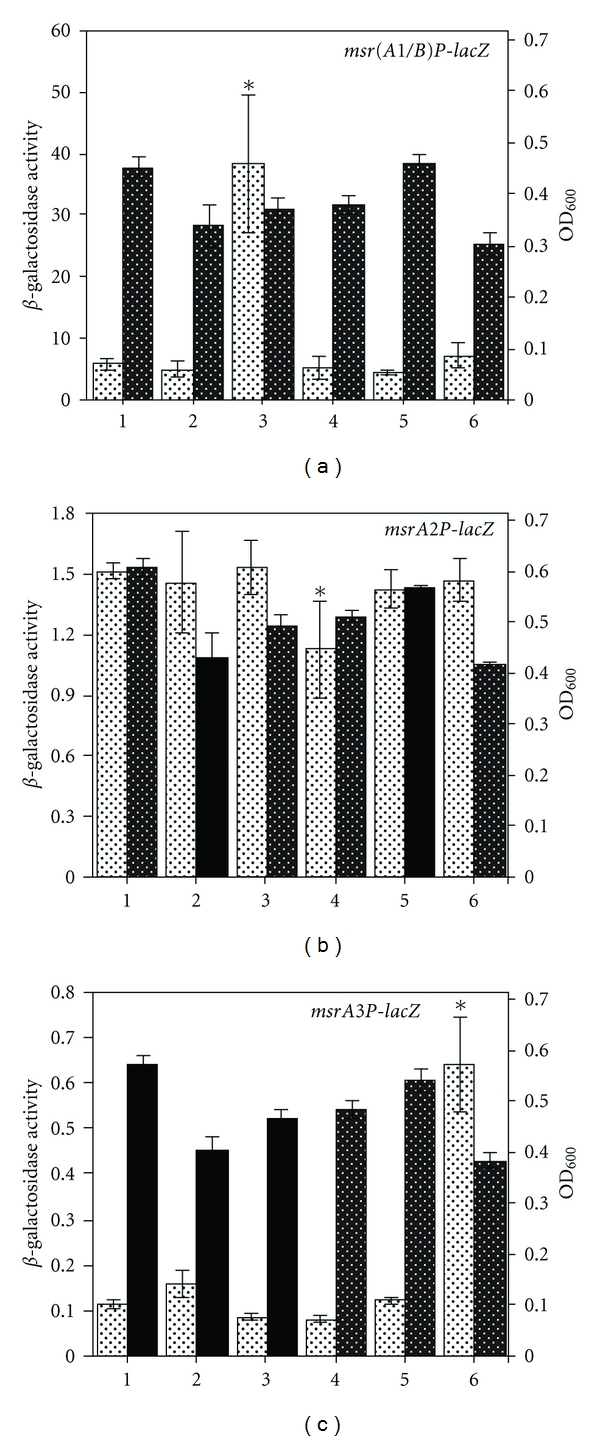
Expression of the *msr(A1/B)*, *msrA2*, and *msrA3* loci in *S. aureus* SH1000 under different environmental stress conditions. Cultures of *S. aureus* SH1000 *msr(A1/B)P-lacZ*, *msrA2P-lacZ*, and *msrA3P-lacZ* reporter strains were grown to OD_600_ of 0.3 at 37°C and treated separately with H_2_O_2_ (15 mM) (2), oxacillin (1.2 *μ*g/mL) (3), pH 5.0 (4), pH 9.0 (5), and TSB with 1.5 M added NaCl (6) for 1 h. *β*-galactosidase activity (*lighter bar*) and growth (OD_600_) (*darker bar*) were subsequently determined. *β*-galactosidase activity and growth of cells in TSB control are represented in *bars 1*. Values indicate averages of data from three independent experiments ± standard deviation (SD).

Expression of *msr* genes in *S. aureus* was investigated under various stress conditions. In these studies, the expression of *msrA1/B* gene locus was significantly increased (~6.5-fold) (Figure 3(a)) in the presence of oxacillin, an observation consistent with prior findings [23–25]. No significant change in the expression of *msrA1/B* gene locus was observed under oxidative, alkaline, acidic, or osmotic stress conditions (Figure 3(a)). None of these stress conditions caused any increase in *msrA2* expression (Figure 3(b)). Expression of *msrA2*, in fact, was significantly repressed under acidic pH (Figure 3(b)). Studies utilizing *msrA3* reporter strains demonstrated an approximately 5.5-fold increase in *msrA3* expression under osmotic stress. Other stress conditions did not significantly affect *msrA3 *expression in *S. aureus *(Figure 3(c)).

### 3.4. Expression of *S. aureus msr* Genes under Chemically Induced Oxidative Stress Conditions

**Figure 4:**
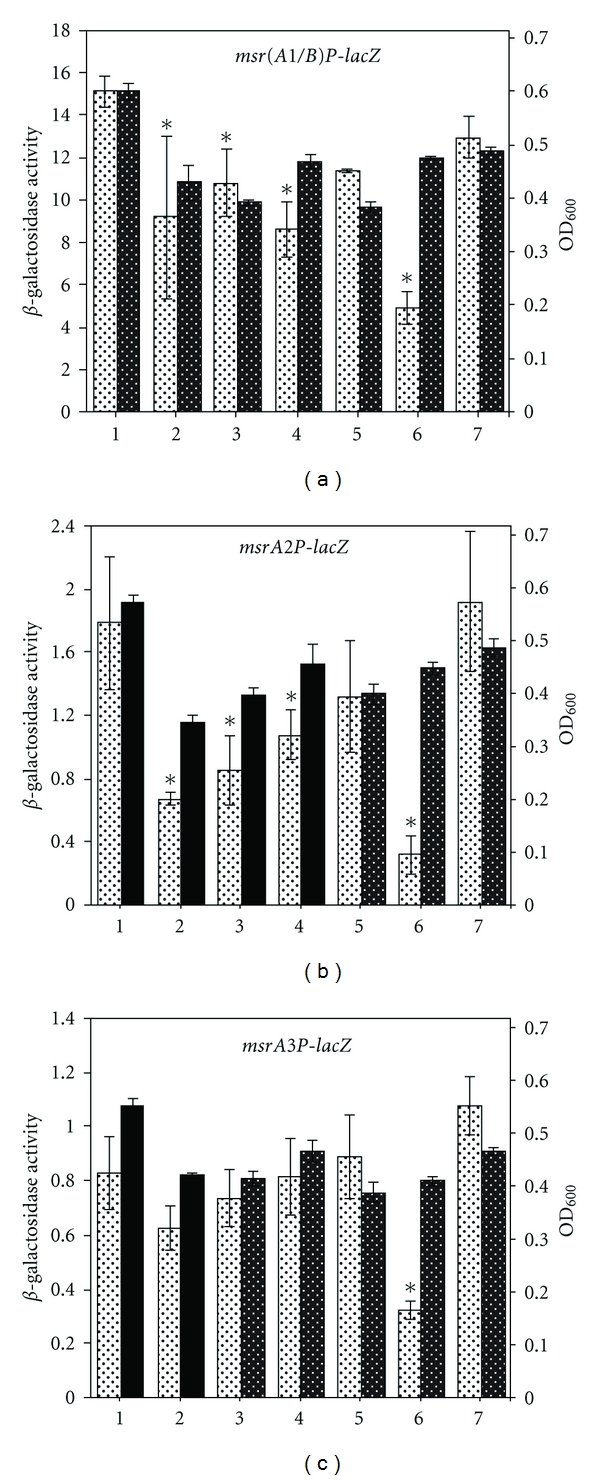
Expression of the *msr(A1/B)*, *msrA2*, and *msrA3* loci in *S. aureus* SH1000 in the presence of different oxidizing chemical agents. Cultures grown to OD_600_ of 0.3 at 37°C were treated separately with the following stresses for 1 h: diamide (5 mM) (2), N-ethylmaleimide (NEM) (0.05 mM) (3), methyl viologen (MV) (20 mM) (4), menadione (MD) (0.05 mM) (5), cumene peroxide (CuOOH) (0.0125%) (6), and sodium nitroprusside (SNP) (5 mM) (7). *β*-galactosidase activity (*lighter bar*) and growth (OD_600_) (*darker bar*) were subsequently determined. *β*-galactosidase activity and growth of cells in TSB control are represented in *bars 1*. Values indicate averages of data from three independent experiments ± standard deviation (SD).

Expression of *msr* genes was determined in actively growing *S. aureus* cells that were exposed for 1 h to various chemicals to induce oxidative stress. The concentration utilized was selected for their ability to show relatively slower growth of treated cultures compared to untreated culture. In these experiments, contrary to the expectations, chemically induced oxidative stress did not induce the expression of any of the *msr* genes (Figures 4(a), 4(b), and 4(c)). Many of these chemicals, most notably, cumene hydroperoxide, methyl viologen, diamide, and NEM, repressed the expression of the *msr* genes to a significant level (Figures 4(a), 4(b), and 4(c)).

## 4. Discussion

Survival of *S. aureus* under various environmental stresses is a key determinant of its pathogenicity. During colonization and invasion of a host, staphylococci are continuously exposed to toxic conditions. Following *S. aureus* invasion, the host responds by recruitment of polymorphonuclear leukocytes and macrophages to the site of infection so that they can ingest the staphylococci. Uptake of bacteria triggers oxygen-dependent microbicidal pathways in the phagocytic cells that generate reactive oxygen species (ROS) such as hydrogen peroxide, hydroxyl radicals, singlet oxygen, and hypochlorous acid [4]. Degradation of phagocytosed bacterial cells in lysosomes is also facilitated by its acidic environment [28]. To defend itself against the oxidative stress of ROS from neutrophils, *S. aureus* has several strategies in place that enables it to successfully colonize and survive in the host. *S. aureus *produces antioxidant enzymes such as superoxide dismutases that convert superoxide anion to hydrogen peroxide, catalase that converts hydrogen peroxide to water and oxygen, alkyl hydroperoxide reductases that detoxify hydrogen peroxide, peroxynitrites and hydroperoxides, and the carotenoid pigment staphyloxanthin that is also involved in the detoxification of ROS [29, 30]. In addition, *S. aureus* also contains methionine sulfoxide reductase enzyme system, which has been shown to be protective from oxidative stress [28].

*S. aureus* produces four Msr enzymes. In this study, we examined the strengths of the three *msr* promoters: *msrA1/B* promoter that drives the transcription of *msrA1* and *msrB* genes, *msrA2* promoter that drives the transcription of *msrA2* gene, and *msrA3* promoter that drives the transcription of *msrA3* gene. *β*-galactosidase activity analysis of *msr* reporter strains revealed that the expression of *msr *in *S. aureus *is growth phase dependent. The expression of *msrA1/B* locus is highest during the stationary phase of growth, whereas the expression of *msrA2* and *msrA3* was higher during the early-to-mid exponential growth phase. Similar stationary-phase-induced expression of *msr* genes has been documented in *E. coli *[20], *Helicobacter pylori *[19], and *Xanthomonas campestris pv. phaseoli *[31]. Overall, the expression of the *msrA1/B* locus in *S. aureus* was observed to be much higher compared to the *msrA2* and *msrA3 *genes. During exponential growth phase, high levels of antioxidant enzymes minimize the intracellular accumulation of oxidants [31], thus offering a likely explanation for lower expression of the more active *msrA1/B *locus during this stage of growth in *S. aureus*. During the stationary phase, nutrient limitation, accumulation of toxic metabolic byproducts, such as ROS, and decreased activity of antioxidant enzymes, such as catalase and superoxide dismutase, increase the likelihood of oxidative damage to cells [31, 32]. The increased expression of *msrA1/B* would serve to alleviate the oxidant-induced damage during the stationary phase. This phenomenon is similar to the induction of MsrA in stationary phase observed in *E. coli* [20].

The expression of *msr* genes was also studied under a variety of stress conditions. This study showed that oxacillin and osmotic stress induced the expression of *msrA1/B* gene locus and *msrA3 *expression, respectively. Besides oxacillin (*msrA1/B*) and salt (*msrA3*), other stress conditions tested in this study had no impact on the expression of *msr *genes. The increased expression of *msrA1/B* in response to oxacillin has previously been demonstrated in *S. aureus *[23–25]. *S. aureus* is one of the most osmotolerant pathogens capable of growing in medium containing upto 3.5 M NaCl [33]. Osmotic stress results in the shrinkage and decreased turgor pressure in bacterial cells. In response, bacteria restore turgor pressure by accumulating osmoprotective solutes, such as glycine betaine, choline, proline, and taurine. It has been previously shown that an exposure of exponentially growing *S. aureus* cells to 2.5 M NaCl significantly increased cell size, and the normal cell size was subsequently restored by the addition of glycine betaine. In addition, muropeptide analysis revealed significant alteration in the morphological structure of cell wall in the presence of NaCl [34]. Under normal conditions, peptidoglycan layers of *S. aureus *cell wall are cross-linked via pentaglycine bridges to provide strong structural framework [3]. However, the cells exposed to NaCl exhibited altered glycine content in the pentaglycine and reduced cross-linking. These structural abnormalities were corrected by glycine betaine [34]. It is, therefore, plausible that the increased expression of *msrA3* under osmotic stress may be related to maintaining cell wall integrity in *S. aureus*. This response would seem to be analogous to the expression of *msrA1/B* in the presence of cell wall-active antibiotics. However, more work needs to be done to fully understand the significance of *msrA3 *and its induced expression under osmotic stress in *S. aureus*.

Induced expression of *msr* genes has been observed in many bacteria under various stress conditions. In *E. coli*, depletion of glucose or nitrogen in the growth media led to a three- to four-fold increase in MsrA activity [20]. Cells exposed to peroxide, peroxynitrite, or dipyridyl (iron-chelator) stress showed a 3-fold *msr* induction in *H. pylori *[19]. Various oxidizing chemicals such as menadione (10-fold), tert-butyl hydroperoxide (6-fold), H_2_O_2_ (3-fold), and N-ethylmaleimide (2-fold) induced *msrA* expression in *Xanthomonas campestris pv. phaseoli* [31]. In *Streptococcus gordonii*, an increase in pH (6.2 to 7.3) induced *msrA* expression [35]. Chemical stress of phenol or chlorophenol induced *msrA* expression by 4-fold and 5-fold, respectively, in the soil bacterium *Ochrobactrum anthropic *[22]. In *Bacillis subtilis*, paraquat, a superoxide generating chemical, induced *msrA* expression by 3.5-fold [36].

The lack of overall *msr* induction in *S. aureus* under oxidative stress was surprising considering that such conditions have been shown to induce the expression of *msr* genes in other organisms. However, it has been speculated that even if *msr* genes are not induced in response to oxidative stress in some species, these gene products are still required to ensure appropriate survival under stress [12]. This was illustrated in *E. coli*, where the oxidizing agents, H_2_O_2_ and paraquat, failed to induce *msrA *expression. However, disk-inhibition studies on solid medium revealed significantly increased growth inhibition of *msrA* mutants in response to H_2_O_2_ [20]. Similarly, a mutation in *msrA1 *rendered *S. aureus* more susceptible to H_2_O_2_ stress, but no induction of *msrA1/B* was noticed on exposure to H_2_O_2_ [13, 16].

In *S. aureus,* the basal level of *msr* expression is probably sufficient to protect the cells from oxidative damage. Alternatively, other stress responsive genes may be able to respond more efficiently to the stress conditions tested in this study, thus bypassing the need for an induction of the *msr* genes. In microarray experiments, at least 25 stress-related genes were upregulated in *S. aureus* upon exposure to ROS. Some of these genes encode enzymes such as catalase, thioredoxin, thioredoxin reductase, superoxide dismutase, alkyl hydroperoxide reductase, and glutathione peroxidase [37]. Nitric oxide produced from sodium nitroprusside reacts with oxygen or superoxide to generate reactive nitrogen species that attack thiols, metal centers, and macromolecules. Proteomic analysis showed that in response to nitric oxide stress in *S. aureus*, a total of 35 proteins were synthesized in elevated amounts [38]. Another study showed a differential regulation of 638 staphylococcal genes in response to nitrosative stress caused by sodium nitrite [39]. Transcriptomic analysis of *S. aureus* in response to hydrogen peroxide-induced oxidative stress revealed differential expression of 343 genes after 10 min and 20 min exposure [40]. Altogether, these results suggest that the induction of additional oxidative stress response genes prevents ROS-induced damage in *S. aureus*.

In summary, the findings of this study suggest that the expression of the *msrA1/B* locus is highest during the stationary growth phase while the expression of *msrA2* and *msrA3* is highest during the early- to mid-exponential phases of growth. The *msrA1/B* locus is under the control of a more powerful promoter compared to *msrA2* and *msrA3* gene promoters. The expression of *msrA1/B* locus is significantly induced by oxacillin, while the expression of *msrA3* is significantly increased in response to osmotic stress. As the oxidative stress conditions did not affect *msr* gene expression, it would be of interest in the future to see if *S. aureus msr* mutants (*msrA1*, *msrB*, *msrA1*:*msrB*, *msrA2*, *msrA3, *a complete *msrA,* or a complete *msr *mutant) show any differential sensitivity to oxidative stress or other stress conditions, which is currently under investigation.

## Figures and Tables

**Table 1:** Bacterial strains and plasmids used in this study.

Strain or plasmid	Characteristics	Reference
Strains		
*S. aureus* SH1000	*S. aureus* strain 8325-4 with functional RsbU	[41]
*S. aureus* RN4220	A restriction minus derivative of *S. aureus* 8325-4	[42]
VKS1009	SH1000 with *msrA1/B*-*lacZ *integration (Erm^r^)	This study
VKS1010	SH1000 with *msrA2*-*lacZ *integration (Erm^r^)	This study
VKS1011	SH1000 with *msrA3*-*lacZ *integration (Erm^r^)	This study
*E. coli* JM109	*recA1 supE44 endA1 hsdR17 gyrA96 relA1thi *Δ(*lac-proAB*) F′(*traD36proAB^+^lacl^q^ΔM15*)	[43]

Plasmids		
pGEMT	Cloning vector for *E. coli* (Amp^r^)	Promega
pAZ106	*lacZ* fusion vector (Amp^r^, *E. coli*; Erm^r^, *S. aureus*)	[27]
pGEMT-*msrA2P *	pGEMT containing the *msrA2* promoter fragment	This study
pGEMT-*msrA3P *	pGEMT containing the *msrA3* promoter fragment	This study
pAZ-*msrA2P *	pAZ106 containing *msrA2* promoter-*lacZ* fusion	This study
pAZ-*msrA3P *	pAZ106 containing *msrA3* promoter-*lacZ* fusion	This study

Erm^r^: erythromycin resistant, Amp^r^: ampicillin resistant.
